# Analysis of Eye and Head Tracking Movements During a Puck-Hitting Task in Ice Hockey Players, Compared to Wrestlers and Controls

**DOI:** 10.11621/pir.2024.0305

**Published:** 2024-09-15

**Authors:** Irina S. Polikanova, Danila D. Sabaev, Natalia I. Bulaeva, Elizaveta A. Panfilova, Sergey V. Leonov, Grigory S. Bugriy, Boris I. Bespalov, Anna P. Kruchinina

**Affiliations:** a *Federal Scientific Center for Psychological and Interdisciplinary Research, Moscow, Russia*; b *HSE University, Moscow, Russia*; c *Lomonosov Moscow State University, Russia*

**Keywords:** hockey, virtual reality, eye movements, eye tracking, eye-movement strategies

## Abstract

**Background.:**

The study of eye-movement strategies of athletes of various disciplines and skill levels is highly significant for sports psychology, since the results can be used in training to improve performance. Such studies are extremely scarce for ice hockey.

**Objective.:**

To determine successful eye-movement strategies for ice hockey players compared to wrestlers and controls (non-athletes) during puck-hitting tasks of various degrees of difficulty, using virtual reality.

**Design.:**

The study involved 31 participants (male), including 13 ice hockey players (age 20 ± 2.5), 9 wrestlers (age 19 ± 1.9), and 9 controls (age 19 ± 1.3). We used a pre-developed VR-PACE technology that simulates an ice rink in virtual reality (VR). The task was to hit pucks. VR was presented via the HTC Vive Pro Eye with a built-in eye tracker (100 Hz). We analyzed the parameter that reflected the share of puck presence in one of selected retina areas (0–5°, 5–10°, 10–15°, 15–25°, 25–35°) of the left and right eyes and the head.

**Results.:**

Ice hockey players exhibited longer puck-tracking using both the near periphery (5–15°) and central retinal area (0–5°). Puck speed had minimal impact on eye-movement strategies, and the visual focus on these areas remained consistent regardless of task type. For both wrestlers and controls, visual fixations in the central retinal area increased when tracking the puck without a motor response, likely leading to higher energy consumption and sensory fatigue.

**Conclusion.:**

The optimal eye-movement strategy involves parafoveal tracking in the near periphery (5–15°) and partial foveal tracking (0–5°), allowing for better object information retention and efficient puck trajectory tracking with reduced energy expenditure.

## Introduction

Ice hockey is known for demanding not only great physical condition from its athletes, but also well-developed cognitive abilities and psychological stability, since players need to simultaneously track the puck, read the team and the field situation, control timing and coach instructions (Bugriy et al., 2022; Leonov et al., 2022; Polikanova, Leonov, et al., 2022; Polikanova, Yakushina, et al., 2022). This, in turn, might testify to better development of their sensory abilities — primarily visual system and sensorimotor coordination, which ensure smooth operation of the complex coordinated movements required for ice hockey. This implies that analyzing eye movements and eye-movement strategies in ice hockey may be relevant for proficiency diagnostics and used to train beginners (Panchuk et al., 2017). Moreover, several authors discuss the possibility of improving perceptual skills in ice hockey (Panchuk et al., 2017). There are currently very few studies of this.

### Eye Tracking in Sports

Eye-movement strategies are important for a number of sports (Hausegger et al., 2016; Krabben et al., 2022; Lafont, 2008; Iakushina et al., 2024), including ice hockey (Panchuk et al., 2017; Panchuk & Vickers, 2006).

Optimal coordination of perception and action is critical for successful performance of sports tasks (Tambovsky, 2003). Foveal vision is characterized by high visual acuity. It is realized in a small retinal area that can be compared to the size of one’s thumbnail (up to 5° of visual angle). But as the distance from this area increases, visual acuity decreases, and at eccentricity (the angular distance from the fovea — all visual information outside of this foveal area) of 40°, information becomes blurred by 90%. Despite the low acuity of peripheral vision, a great number of rod cells provide high motion sensitivity in this retinal area (Strasburger et al., 2011).

To process information with high visual acuity, humans use movements of their body, head, and eyes to shift the fovea to specific areas of interest. These movements are divided into saccades, smooth pursuit eye movements, vergence, and vestibular eye movements. Humans mainly rely on saccades, which are rapid eye movements with speeds up to 500°/s (Rayner, 1998). When performing saccades, sensitivity to visual information decreases, a phenomenon known as saccadic suppression (Binda & Morrone, 2018). In contrast, during smooth-pursuit eye movements, visual-information sensitivity is similar to fixations, meaning periods of relative eye stillness, but only occurs when the eyes follow an object (Spering et al., 2011). Therefore, the movement of the fovea is costly due to information loss. To avoid these costs, it is reasonable to assume that, especially in sports, where acting in complex environments with strict timing and precision requirements is necessary, eye movements are adjusted depending on the task.

Therefore, the operability of foveal and peripheral vision in sports is determined by the current sport situation and task — if there is one or more relevant stimulus; if the task is to aim or track, etc. (Klostermann et al., 2020).

During fixations, the objects that come within the central visual area that provides pattern vision (approximate diameter .5 mm, approximately 2° of visual field) are perceived and recognized. To perceive and analyze a complex situation requires numerous rapid eye movements (saccades) to move the point of fixation from one object to another. Also, as noted above, the processing of information during saccades is suppressed, causing the loss of essential information during gaze shift. This, in turn, may be crucial for fast-paced and dynamic sport situations (Binda & Morrone, 2018). That is why certain skills are necessary in sports. One of those skills is the optimization of eye movements, considering the sport task and the capabilities of foveal and peripheral vision. Another skill is the ability to process incoming visual information regardless of the visual field area (central or peripheral) from which this information was received. These skills are developed through training, and set professional athletes apart from beginners (Mann et al., 1996; Panchuk et al., 2017).

The ability to focus visual attention on objects using peripheral vision, i.e., without fixing the gaze on them, is an essential skill for an athlete. This skill is especially useful when there is more than one relevant object in sight and/or when the time needed for decision-making is limited. Peripheral vision is more beneficial for simultaneous tracking of multiple moving objects and for detecting response-demanding alterations in those objects (Vater et al., 2017).

Skilled athletes may use different strategies to optimize their eye-movement activities in any given situation. For instance, 1) gaze fixation on a single point (anchoring) and distribution of visual attention via peripheral vision to track multiple objects; 2) focus and concentration of visual attention on one relevant object via predominantly foveal and parafoveal vision; 3) gaze orientation along a certain axis between several relevant stimuli via peripheral vision and the possibility of shifting the gaze from one object to another with minimal consumption of time and energy (Klostermann et al., 2020; Vater et al., 2020).

When the vision field is restricted, skilled athletes are capable of analyzing the situation and making proper decisions based on visual information regardless of the visual field area (central or peripheral) from which this information was received (Ryu et al., 2013).

### Eye-Movement Strategies in Various Sports

In martial arts (such as Kung Fu and Taekwondo), gaze fixation on a particular part of the opponent’s body (“anchoring”) is the most effective way to optimize the use of peripheral vision. Choosing where to anchor your gaze depends on the particular fight situation, key (motor) signals, and possible loss of visual information during saccades. Highly skilled athletes use peripheral vision to assess their opponent’s attacks, switching visual attention instead of saccadic gaze shifts (Hausegger et al., 2016).

Before their fight, professional judoka gaze primarily at the opponent’s chest area. This helps them monitor their opponent’s hands by peripheral vision (Krabben et al., 2022). Beginners, however, are more likely to gaze at the opponent’s sleeves and show a higher number of quick gaze fixations (Piras et al., 2014).

The comparison of world-class tennis players to their non-world-class colleagues revealed a pivotal difference in their eye-movement patterns during ball bounce. Before returning the ball, world-class tennis players shifted their gaze to the contact area (where the racket meets the ball) and fix it there until they hit (Lafont, 2008).

The eye-movement strategy chosen by cricket players while batting affects the success of ball reception. Professional players shift their gaze faster to the expected bounce point of the ball after their opponent’s shot and track it for 100–200 ms after it bounces off the ground. This helps the batter to predict the ball’s trajectory in a short time (about 500 ms) (Land & McLeod, 2000). At the same time, two predictive saccades (expected ball bounce spot and bat-to-ball contact spot) are distinctive features of the eye-movement strategy of world-class cricket players (Mann et al., 2013).

Professional baseball batters coordinate their head rotation with the ball’s movement, ensuring that the ball is constantly moving relative to their head. World-class batters use specific eye-movement strategies, usually relying on two predictive saccades to anticipate (a) ball bounce spot and (b) spot of bat-to-ball contact. This allows them to aim their gaze at the ball at the time of impact (Mann et al., 2013).

### Visual Areas

Despite its lower visual acuity as compared to the fovea, the periphery performs an essential function in visual perception. The processing of visual signals received by foveal and peripheral retinal areas are closely interconnected both during fixations and saccades. Such integration of central and peripheral stimuli contributes to continuity and reduces the uncertainty of perception of the environment (Stewart et al., 2020).

The boundaries between the foveal area and periphery are nominal — there are no clear borders between them. This uncertainty echoes in the vagueness of the vocabulary used in studies of central and peripheral vision. How should we label the central field of vision and how the peripheral?

Perimetry defines the central visual field as an area of approximately 60° in diameter (vision area shared by both eyes). Anything beyond this area could be considered the peripheral visual field. On the other hand, the retina has a specific structure called the macule. It is about 17° in diameter (comprising the foveola of about 1° in diameter, the fovea of about 5.2° in diameter, and the parafovea and perifovea of about 5–9° and 9–17° in diameter, respectively). The macule is the central retinal area and is responsible for high-resolution color vision. So, we can attribute everything that falls in the macule area to the central visual field (of each eye). Meanwhile, the foveal visual area is a region of 2° surrounding the fovea centralis. Everything beyond those borders is considered the peripheral visual field (Strasburger et al., 2011).

Five main areas were also nominally identified in the study of perceptual abilities of visual field periphery. This subdivision broadly reflects the visual field structure conditioned by specific features of retinal structure. The areas differ by their ability to process different stimuli and solve different types of tasks (detection, recognition, identification, classification of test objects):

*central* (±2.50°) — the highest level of detection, recognition, identification, and classification of objects shown for a short time (several tens of milliseconds);

*near periphery* (±2.5–15°) — comparatively high level of detection, recognition, identification and classification of objects; possibility of anticipating their changes;

*middle periphery* (±15–25°) — limited capacity to recognize and identify short-term objects; distinct classification difficulties;

*far periphery* (±25–35°) — good detection, but poor identification, recognition, and classification of objects;

*extreme periphery* (above ±35°) — detection only (Barabanschikov, 1990; Strasburger et al., 2011).

This classification served as the basis for the present study. Our aim was to identify successful eye-movement strategies in ice hockey players compared to other athletes (freestyle wrestlers) and controls during puck-hitting tasks of various degrees of difficulty, using virtual reality.

Given the theoretical analysis, we developed the following hypotheses:

1) When comparing missed (“goal yes”) and save pucks (“goal no”), the successful eye-movement strategies are characterized by the prevalence of visual attention focus on pucks in the central retinal area and near periphery;
2) Ice hockey players significantly differ from other groups of subjects in terms of the puck-holding dominance in the central retinal area and near periphery;
3) As puck speed increases, the group differences in visual puck retention in the central retinal area and near periphery will increase;
4) Eye-movement strategies in the no-motor-response puck-viewing task (block 5) differ from eye-movement strategies in the motor-response tasks (blocks 1–4).

## Methods

### Participants

The study involved 31 participants (men), including 13 professional ice hockey players (age 20 ± 2.5; average training experience 14.18 ± 3.8 years; different skill levels (Candidate Master of Sports — 3, 1st Senior Category — 1, 2nd Senior Category — 6, 3rd Senior Category — 1, 1st Junior Category — 2), and 9 freestyle wrestlers (age 19 ± 1.9; average wrestling experience 8 ± 6.10 years, Candidate Master of Sports — 1, Senior Category — 2, Junior Categories — 4), 9 controls (age 19 ± 1.3). Women were excluded due to the necessity for taking the menstrual cycle into account.

All subjects volunteered for the study following a pre-signed consent and prior approval from the Ethics Committee of the Russian Psychological Society (March 2021) in line with the Declaration of Helsinki.

### VR-PACE Virtual Reality Technology

The study involved the previously developed original VR-PACE (**VR** Technology for training **P**uck hitting **A**nd ho**C**key skill **E**ffectiveness) using virtual reality (HTC Vive Pro Eye), aimed at diagnosing and analyzing the skill level of ice hockey players, as well as their training. For a more detailed description, please see Polikanova, Leonov et al. (2022). Before the experiment starts, the subject puts on ice hockey equipment — shin guards, skates, gloves, and a virtual reality helmet. The subject stands on special plastic ice. He holds a stick that matches the stick in the virtual environment. Pucks are presented in blocks that differ by level of difficulty. There are a total of five blocks; the difficulty increases from block 1 to block 4. Block 1 is the easiest (speed 60–80 km/h, long distance to the puck — 18 m). Block 2 is more difficult (speed 60–80 km/h and 100 km/h, average distances to the puck are greater — 12 and 18 m). Block 3 is for a medium skill level; it is a challenging mode with high speeds (up to 170 km/h) and any distances, including close ones (6, 12 and 18 m). Block 4 is the most difficult (pucks are presented in a series of two consecutively with a 1 s interval). Block 5 suggests that the subject does not hit the pucks, but only closely observes and tracks them.

The virtual environment has its own limitations and strengths. In our work, we formalize the concept of a person’s visual strategy when interacting with a moving puck by analyzing the distribution of the puck’s time spent in different zones of the person’s visual field. By transitioning from describing the movement of the eye to analyzing the formalized visual strategy, we gain the ability to quantitatively compare different cases.

The primary advantage of a virtual environment lies in its capacity to provide comprehensive and detailed information about the state of the environment and to isolate external distracting factors.

### HTC Vive Pro Eye

The Vive Pro Eye is a high-end virtual reality (VR) headset developed by HTC for professional and enterprise use. It builds upon the foundation of the original Vive Pro, with the addition of integrated eye-tracking technology. Similar to the positional data (Leonov et al., 2022) of the trackers, eye movement data exhibits temporal irregularity. This complicates the analysis, but does not make it impossible. Moreover, the recording frequency is not high, around 100 Hz, so the irregularity further reduces the ability to determine the type of movement and accurately reconstruct trajectories.

It is worth noting that the data recorded by the VivePro Eye system requires additional recalculation of the eye coordinates into a common coordinate system. It was also necessary to reconstruct the information about the position of the origin of the eye coordinate system relative to the tracked point on the helmet.

### Investigated Characteristics

As the primary variable for analysis, we chose the position of the puck’s image relative to the player: in which part of the field of view the puck was located. During the recording, the information on the exact puck coordinates, head coordinates and orientation, eyes position and orientation was captured. The entire described dataset was recorded at similar frequencies (100 Hz). Based on this data, it was determined which part of the retina and which part of the person’s field of vision the observed object — the puck — was located in.

Visual stimuli (pucks) were presented for varying durations — “lifetimes”. The difference in their lifetime stemmed from differences in initial conditions such as speed and distance from the goal. Direct comparison of relative trajectories is not feasible due to significant variation in initial conditions. When observing the puck, the average eye velocity over .01-second intervals does not show significant deviations from values typical for slow eye movements. Therefore, we assume that during the puck’s flight, the eye performs a task of fixation and tracking. Consequently, we suggest that throughout the observed time interval, the retina is consistently recognizing the image of the puck.

When observing the puck, the expected value of speed (at a sampling rate of 100 Hz) was 50 °/s with a standard deviation of approximately 30 °/s (the averaging was performed over a sample of all pucks). It is important to note the significant influence of individual trials with high average speeds, which result from both measurement errors and the presence of relatively quick shifts in gaze. Considering these values, we will assume that the puck was perceived by the person throughout its entire duration.

We proposed using a vector of parameters to describe the visual interaction with the puck. The total duration is segmented into intervals. During each interval, the puck remained within one of the specific areas of the player’s visual field. To obtain dimensionless time, the time the puck spends in each area of the player’s visual field is divided by the puck’s lifetime total duration.

### Why Don’t We Use Classical Characteristics?

Classical metrics include characteristics of fixations and saccades, though there is often inconsistency in defining these states (Hessels et al., 2018). “Fixation” and “saccade” detection depends on the eye-tracking sampling rate, because definitions are often based on the concept of eye-rotation velocity. For the term “saccade”, two understandings can be distinguished: physiological — rapid eye movement corresponding to an uncorrectable control pattern during execution (Holmqvist et al., 2012; Kruchinina & Yakushev, 2018), and non-physiological, which is equal to “no fixation”. In mathematical terms, we should talk about periods of average fast movement and average slow movement. A mandatory parameter in this case is the averaging time, which is inversely related to frequency. For example, at 100 Hz maximal average eye velocity is approximately 500°/s, if the eye makes a saccade by amplitude 50°. As is known, the maximal velocity will be approximately 800°/s. An additional glissade cannot be detected. So, if we have data in a sample range less than 250 Hz, it is preferable to use an alternative approach.

### Meaning of Investigated Characteristics

A similar measurement, such as the angle of the puck relative to the head direction, can be used to assess visual strategy. This indicates to what extent a person achieves visual objectives through eye movements versus head movements. If the eyes only make small scanning movements, then head movements essentially determine the gaze direction. On the one hand, the eyes may only perform small scanning movements, which do not significantly influence the retinal area used for perceiving a moving object. On the other, the eyes can precisely position the image of the object on the desired retinal area, indicating the sensitivity of the chosen parameter vector to the implemented visual strategy.

### Analysis of Data and Eye-Movement Strategies

Benchmarking was performed using Jamovi 2.4.1. A test for normality by Shapiro-Wilk criterion showed that all parameters are not normally distributed; therefore, it was decided to apply the Mann-Whitney *U* test. The non-parametric Wilcoxon test (α = .05) was used for intragroup comparisons. The Kruskal-Wallis criterion was used for analysis of variation (One-Way ANOVA).

To analyze eye-movement strategies, we used the parameters of angles between (a) left eye and puck, (b) right eye and puck, and (c) head and puck.

As stated earlier, we used the following angle values that characterize the areas of central and peripheral vision: 0–5°, 5–10°, 10–15°, 15–25°, 25–35°.

The gaze share in each angle area was calculated. This parameter was calculated for each of the parameters described below:

− for 4 speed ranges (velocity ranges were calculated as puck lifetime, which depended on distance and initial velocity): very fast (.157 s to .365 s), fast (.365 s to .573 s), slow (.573 s to .781 s), very slow (.781 s to .989 s);
− for each group (ice hockey players, wrestlers, controls);
− for missed pucks (“goal yes”) and saves (“goal no”);
− for each of the five blocks.

## Results

### Analysis of Missed Pucks and Saves

Statistical analysis for the left and right eye values showed that eye-movement strategies in the case of successful saves are marked by a prevalence of focusing visual attention on pucks in the central retinal area (0–5°) as well as the near periphery (5–15°), compared to missed pucks (*Table 1*). The statistical analysis of head parameters (angle between head and puck) showed that in the case of successful saves, visual fixations in the near (5–15°) and middle periphery (15–25°) prevailed, compared to missed pucks (*Table 1*). Thus, the data allows us to accept hypothesis 1, stating that in successful saves, visual focusing will prevail when the puck is tracked by central retinal areas as well as the near periphery (for eyes).

**Table 1:** Results of Statistical Analysis of Eye-Movement Strategies for Successful Saves and Missed Pucks

Angles	Goal	LEFT EYE					RIGHT EYE					HEAD				
		Mean	*SD*	*P*	*U*	Cohen’s *d*	Mean	*SD*	*P*	*U*	Cohen’s *d*	Mean	*SD*	*P*	*U*	Cohen’s *d*
0-5°	Yes	**.0890**	**.172**	<.001***	**74.765**	**.1969**	**.0948**	**.180**	<.001***	**74.146**	**.2035**	.0348	.105	.420	91.060	.0218
	No	**.1840**	**.258**				**.1904**	**.262**				.0382	.123			
5-10°	Yes	**.1622**	**.217**	<.001***	**74.028**	**.2048**	**.1651**	**.220**	<.001***	**74.361**	**.2012**	**.0931**	**.162**	<.001***	**80.242**	**.1380**
	No	**.2524**	**.270**				.2572	.279				.1457	.227			
10-15°	Yes	**.1309**	**.196**	.006**	**83.229**	**.1059**	**.1312**	**.200**	.003**	**82.536**	**.1134**	**.1520**	**.208**	<.001***	**77.096**	**.1718**
	No	**.1740**	**.237**				**.1763**	**.238**				**.2103**	**.237**			
15-25°	Yes	.1792	.235	.532	90.794	.0247	.1739	.230	.707	91.709	.0148	**.2633**	**.285**	<.001***	**73.297**	**.2126**
	No	.1937	.257				.1877	.250				**.3828**	.324			
25-35°	Yes	.0432	.111	.390	90.439	.0285	.0418	.101	.540	91.199	.0203	.0418	.101	.540	91.199	.0203
	No	.0484	.117				.0447	.113				.0447	.113			

*^1^ Goal: yes — goal; no — saved puck* Notes. * p < .05, ** p < .01, *** p < .001

To refine the results even more, a nonparametric one-way ANOVA (Kruskal-Wallis criterion) analysis of variance was performed, which showed significant intergroup differences only for goals for the near periphery (5–10°) for both eyes (*Table 2*). For the head, the nonparametric one-way ANOVA analysis of variance showed no significant inter-group differences.

**Table 2:** Significant Statistical Differences (Nonparametric One-Way ANOVA) Between Different Groups on Visual Attention Focusing on Pucks in the Near Periphery (5–15°) in the Case of Goals

Eye	Sport	Mean	SD	*p*	χ^2^	ε^2^
left	Hockey	.199	.239	.008	9.66	.017
	Wrestlers	.125	.181			
	Controls	.162	.220			
right	Hockey	.193	.230	.006	1.07	.017
	Wrestlers	.127	.187			
	Controls	.171	.230			

Additional Dwass-Steel-Critchlow-Fligner pairwise comparisons analysis showed significant differences for the left eye between ice hockey players and wrestlers (*W* = –4.27, *p* = .007), and marginal means between ice hockey players and controls (*W* = –3.21, *p* = .06); for the right eye between ice hockey players and wrestlers (*W* = –4.52, *p* = .004).

The data allows us to accept hypothesis 2, with several adjustments. Compared to other groups, for ice hockey players it is typical to track the puck longer at the near periphery (5–10°) in the case of goals. For the wrestlers and controls, there is also a primary prevalence of puck-tracking in the central retinal area (0–5°) and the near periphery (5–15°).

For head parameters, the middle periphery (15–25°) dominates in all three groups.

### Speed Analysis of Puck Perception

*Table 3* shows the results of nonparametric one-way ANOVA, which revealed significant differences between groups depending on puck speed. The most significant inter-group differences were observed for the central retinal area (0–5°) of the left and right eyes at both slow and fast speed, and for the near periphery (10–15°) of the head at very slow speed.

**Table 3:** Results of Statistical Analysis of Eye-Movement Strategies at Different Speeds of Puck Presentation

Speed	Eye/Head	Angle	Sport, mean (*SD*)			*p*	χ^2^	ε^2^
			Hockey	Wrestlers	Controls			
VERY SLOW	Head	10–15	.063 (.11)	.0169 (.253)	.239 (.212)	.033	6.795	.2
SLOW	Left eye	0–5	.313 (.285)	.167 (.211)	.219 (.236)	.036	6.622	.046
FAST	Left eye	0–5	.142 (.225)	.0684 (.147)	.0972 (.188)	.010	9.174	.021
	Right eye	0–5	.133 (.2)	.101 (.198)	.0976 (.195)	.042	6.337	.12
	Right eye	5–10	.243 (.257)	.166 (.245)	.222 (.274)	.010	9.145	.021

Additional Dwass-Steel-Critchlow-Fligner pairwise comparisons analysis showed significant differences for *very slow speed* between ice hockey players and controls (*W* = 3.82, *p* = .02).

Additional Dwass-Steel-Critchlow-Fligner pairwise comparisons analysis showed significant differences for *slow speed* between ice hockey players and wrestlers (*W* = –3.54, *p* = .033).

Additional Dwass-Steel-Critchlow-Fligner pairwise comparisons analysis showed significant differences for *fast speed* between:

*For left eye (0–5)*

ice hockey players and wrestlers (*W* = -3.9, *p* = .016),

ice hockey players and controls (*W* = -3.34, *p* = .048).

*For right eye (5–10)*

ice hockey players and wrestlers (*W* = -4.39, *p* = .005).

Thus, we only partially accept hypothesis 3, since significant differences between the groups are observed only in the eyes for slow and fast speeds, and for the head — for very slow speed.

### Analysis of Eye-Movement Strategies with or without Motor Response

To check hypothesis 4, we conducted a statistical analysis, the results of which are shown in *Table 4*.

**Table 4:** Analysis of Eye-Movement Strategies with and without Motor Response

Group	Block	LEFT EYE (0-5°)					RIGHT EYE (0-5°)					HEAD (15-25")				
		Mean	*SD*	*P*	*U*	Cohen's *d*	Mean	*SD*	*P*	*U*	Cohen's *d*	Mean	*SD*	*P*	*U*	Cohen's *d*
ALLGROUPS	Block 1-4	**.123**	**.211**	.007**	**57,863**	**.1203**	**.129**	**.218**	<.001***	**55,618**	**.1544**	.306	.306	.135	60,875	.0745
	Block 5	**.153**	**.209**				**.183**	**.227**				.334	.334			
HOCKEY	Block 1-4	.156	.235	.696	5,526	.0339	.153	.224	.995	5,716		.317	**.313**	.026*	**4,529**	**.2082**
	Block 5	.155	.234				.159	.223				**.289**	**.316**			
WRES-TLERS	Block 1-4	**.0934**	**.178**	<.001***	**2,671**	**.338**	**.121**	**.219**	<.001***	**2,671**	**.338**	.288	.305	.287	3,619	.103
	Block 5	**.195**	**.218**				**.248**	**.239**				.352	.284			
CON-TROLS	Block 1-4	.115	.207	.104	12,238	.1064	**.117**	**.211**	.024*	**11,635**	**.1504**	.307	.299	.214	12,430	.0924
	Block 5	.128	.186				**.160**	**.218**				.352	.287			

Notes. * p < .05, ** p < .01, *** p < .001

Average data for the whole sample demonstrate a significant alteration in eye-movement strategies when switching from a task with motor responses (blocks 1–4) to visual tracking only (block 5). In particular, there is a relevant increase in the share of pucks in the central retinal area (for left and right eyes, but not for the head).

Ice hockey players do not demonstrate such a tendency; they significantly change the head parameters — they decrease.

Thus, hypothesis 4 is accepted with several limitations, since the group of ice hockey players did not show a significant tendency.

## Discussion

The results we obtained are, in general, consistent with data in the literature (Panchuk & Vickers, 2006; Ripoll & Fleurance, 1988). Earlier studies reported the importance of eye-movement analysis as a predictor for performance in cases of early detection and tracking of a still or flying object for different sports (Bard & Fleury, 1981; Ripoll & Fleurance, 1988). Panchuk and Vickers (2006) analyzed eye-movement strategies of goalkeepers when reacting to shots from 5 and 10 m on-ice. They found that success was not determined by puck distance, but rather by location, start and duration of final fixation/tracking gaze (or quiet eye, QE). The relative onset of quiet eye was significantly (p < .001) earlier (8.6%), and the duration was longer on saves (*M* = 8.5%; 952.3 ms) compared to goals (onset 18.86%; *M* = 7.1%, 826.1 ms). The quiet eye was located on the puck/stick during the preparation and execution of the shot in 7.53% of all trials, or on the ice in front of the release point of the puck (25.68%), and rarely on the body of the shooter (2.1%) (as per Panchuk & Vickers, 2006). These results were confirmed by the authors in a recent study (Panchuk et al., 2017).

This data is fully consistent with our results regarding the comparison of successful saves and goals. We demonstrated that in the case of save pucks, visual fixations are dominant in the central retinal area (0–5°) and near periphery (5–15°) for both eyes. Meanwhile, the dominant head-to-puck distance is always slightly greater: in the near and middle periphery (5–25°). Moreover, the most meaningful variations between ice hockey players and other groups are observed in the central retinal area (0–5°). This agrees with data obtained from baseball (Fogt & Zimmerman, 2014) and cricket (Mann et al., 2013). For example, Fogt and Zimmerman (2014) found that Division 1 college baseball players follow the pitched ball with their head throughout the entire pitch trajectory, while their eye moves very little to the end of the pitch trajectory. On average, gaze position matched target position along the entire pitch trajectory. However, eye and head movements were related by a common rule for all subjects (partial suppression of rotational vestibulo-ocular reflex).

Our data also complement the finding of Panchuk (2016) that there is a longer quiet eye on pucks deflected compared to goals. Thus, a longer quiet eye that tracks the central region and near periphery is a professionally relevant parameter. The results allowed us to accept hypothesis 1, that when comparing missed pucks (“goal yes”) and retained pucks (“goal no”), successful eye movement strategies are characterized by a predominant focus of visual attention on pucks in the central retinal area and near periphery. The data obtained allow us to accept hypothesis 2 with several adjustments. Compared to other groups, for ice hockey players it is typical to track the puck longer at the near periphery (5–10°) in the case of goals. Meanwhile, for groups of wrestlers and controls, there is also a primary prevalence of puck tracking in the central retinal area (0–5°) and the near periphery (5–15°).

Successfully performing an interceptive action requires precisely coordinating the movements of an effector (e.g., limb, racquet, or glove) with an approaching target object (Bespalov, 2023; Vickers, 2007; Yakushina et al., 2023). When the object’s trajectory is largely predictable, the flight path can be determined early from the moment of release. In the case of activities that involve less predictable object flight, which occurs during deflection in soccer or ice hockey, perceptual information that completely specifies the point of interception does not emerge until relatively late in the object’s trajectory. As a result, performers must develop perceptual-motor strategies to overcome task constraints specific to their performance environment (Panchuk, 2016).

Our data also enhance the understanding of the role of head movements in interoceptive actions. Our results clearly demonstrate that the dominant head-to-puck distance is about 5–25° for all groups, and most noticeably at slow speeds (puck lifetime — .781 to .989 s). Hockey players in contrast to other groups demonstrate a significant predominance of head-to-puck distance at values 15–25° at slow speeds. It can be assumed that in this way head movements allow the player to optimize visual perception. According to our data, we see that the main range of puck retention by the eyes is from 0 to 15°. Thus, head movements allow the player to provide the most effective visual-motor control for realization of interoceptive actions.

This, in turn, aligns with the findings of other researchers (Bongers & Michaels, 2008; Hayhoe et al., 2012). When catching a ball, moving the head helps adjust the egocentric reference point, meaning the head shifts to maintain a more stable direction of the ball in relation to it. This idea aligns with the understanding that visual-perceptual and visual-motor tasks utilize different kinds of visual information. Specifically, individuals engaged in visual-perceptual tasks collect information from an allocentric perspective (focused on the object in relation to its environment), whereas those performing visual-motor tasks gather information from an egocentric viewpoint (based on their own position) (De Wit et al., 2012; Mann et al., 2013; Milner & Goodale, 1995). Mann et al. (2013) examined the eye and head movements of two of the world’s best cricket players, and found that the batters coordinated their head rotation with the ball’s movement, ensuring that the ball is constantly moving relative to their head. To this end, the ball could be followed if the batters simply moved their head and kept their eyes still.

The results we obtained also greatly contribute to the literature, since we demonstrated the differences in eye-movement strategies when presenting the puck at different speeds, as well as with different tasks (hitting or eye-tracking). Analysis of eye-movement strategies at different speeds of puck presentation showed relevant inter-group distinctions between groups. The results showed that the most significant inter-group differences are observed for the central retinal area (0–5°) of the left and right eyes at slow and fast speed, and for the near periphery (10–15°) of the head at very slow speed.

This allowed us to accept hypothesis 3, that group differences in puck retention in the central retinal region and near periphery would increase with increasing puck velocity.

At the same time, *post hoc* analysis showed significant differences between the group of ice hockey players, wrestlers, and controls (left eye, 0–5°); hockey players and wrestlers (right eye, 5–10°). Ice hockey players were distinguished by a significantly higher gaze share in this retinal area. It should be noted that the “very fast” puck speed corresponds to the maximum puck speed registered in ice hockey (max. registered puck speed in ice hockey history is 177.5 km/h). In other words, such speeds are usually not observed in normal practice. “Very slow” is also not typical for professional hockey, as the speed is usually higher.

Other important data were obtained in comparing eye-movement strategies in the case of a motor-response task (hitting with a stick) and a non-motor-response task (puck eye-tracking). Ice hockey players show no variation in these tasks, whereas the share of the central retinal area tracking is much higher in the other groups in the non-motor response task. This may testify to the high level of sensorimotor coordination of ice hockey players. Other groups do not show this kind of coordination; therefore, it is easier for them to perform the task when a motor system is not involved (only the visual modality is activated). Thus, hypothesis 4 is only partially accepted, because eye-movement strategies in the no-motor-response puck-viewing task (block 5) difer from eye-movement strategies in the motor-response tasks (blocks 1–4), not only for the wrestler and control group, but for the hockey players.

## Conclusion

In summary, the data obtained allow us to accept hypothesis 1 and partially hypotheses 2, 3, and 4.

Efficient eye-movement strategies in ice hockey players include long-term puck tracking, primarily using the near periphery and central retinal areas. The ability to track the puck (especially at high speeds) by the central retinal area is what sets professionals apart from other groups. It is important to note that puck speed rate affects eye-movement strategies, but preferentially for slow and fast speeds (in our work, these speeds correspond to typical speed ranges in ice hockey). The greatest differences between hockey players and the other groups are observed at fast speeds.

Significant differences between hockey players and other groups at very slow speeds are only observed for the head-to-puck parameter in the 15–25° range. It can be assumed that in this way head movements allow the player to optimize visual perception. According to our data, we see that the main range of puck retention by the eyes is from 0 to 15°. Thus, head movements allow the most effective visual-motor control for realization of interceptive actions.

Regardless of the task (hitting or tracking), ice hockey players have almost no change in the visual focus share with dominance in the near periphery (5–10°) and central retinal area (0–5°). The groups of wrestlers and controls had a significant increase in the visual fixations’ share in the central retinal area when they visually tracked the puck without motor reaction. We may conclude that the most effective eye-movement strategy would be the dominance of parafoveal puck-tracking in the near periphery (5–15°), and partially foveal-tracking (0–5°). On the one hand, this prevents the information on the object (puck) from being lost. On the other, it helps track the puck trajectory in a more efficient way, with minimal energy consumption. It appears that a drastic increase in the foveal eye-movement strategy shared in block 5 with wrestlers and controls will lead to high energy consumption and, accordingly, rapid sensory fatigue.

## Limitations

The following are possible limitations to this study. The viewing angle in a VR helmet is limited to 110°; hence it may have affected the parameters of eye movements. The quality of the data could also have been affected by hardware parameters, as well as motion artifacts, sweating, which, in turn, could cause the loss of some data. In addition, the sample was not large. In the case of VR hockey, it was found to be difficult to identify such parameters of eye-movement as fixations and saccades from the primary data obtained for two reasons: 1) low quality of the data obtained, 2) small intervals of stimulus (puck) presentation. Therefore, instead of analyzing fixations and saccades, it was decided to examine the angle magnitude between the subject’s gaze direction and the distance from the subject’s eyes to the puck.

In this paper, eye-tracking analysis was conducted for each eye individually. We propose that future studies should consider averaging the data by eye during the result analysis phase. This approach would help mitigate issues arising from tracking failures in one of the eyes.
